# Biomechanical optimization study of posterior tilt extension stems in the repair of tibial plateau bone defects

**DOI:** 10.3389/fbioe.2025.1688915

**Published:** 2025-11-07

**Authors:** Yong Wang, Ruihu Hao, Lin Guo, Xiaoyu Zhou

**Affiliations:** 1 Affiliated Zhongshan Hospital of Dalian University, Dalian, China; 2 Dalian Medical University, Dalian, China; 3 Dalian Jiaotong University, Dalian, China

**Keywords:** tibial plateau bone defect, posterior tilt, extension stem, biomechanical optimization, finite element analysis, osseointegration, stress distribution

## Abstract

**Background:**

Tibial plateau bone defect represents a pivotal challenge in revision knee arthroplasty, where suboptimal extension stem design predisposes to stress concentration and subsequent prosthesis loosening. Physiological posterior tibial slope (5°–7°) optimizes knee biomechanics, yet bone defects disrupt proximal tibial anatomy, rendering traditional stems biomechanically incompatible. The synergistic optimization of “defect severity-stem length-posterior tilt angle” remains underexplored.

**Methods:**

A finite element model was constructed incorporating three defect areas (20%, 40%, 60%), two stem lengths (40mm, 80 mm), and five posterior tilt angles (0°–10°), yielding 30 experimental cohorts. Under 2450N axial loading, stress distribution (cortical/cancellous bone, prosthesis, sleeve) and bone-prosthesis micromotion were quantitatively evaluated.

**Results:**

All micromotion magnitudes remained below the 150 μm osseointegration threshold. In 20% defects, 40 mm stems with ≤7° tilt mitigated cortical stress concentration; 80 mm stems showed lower micromotion but excessive cancellous stress at 10° tilt. In 40%/60% defects, increasing tilt reduced micromotion (37.3%/45.3% reduction), with 80 mm stems exhibiting superior stability. Extreme tilt (10°) in long stems exacerbated cortical stress and prosthesis load.

**Conclusion:**

Based on the finite element analysis results, this study provides a hypothetical reference for the selection of posterior tilt angles of extension stems in the repair of tibial plateau defects: a posterior tilt angle of ≤7° is suggested for 20% defects when using a 40 mm stem; 7°–10° for 40% defects when using an 80 mm stem; and 5°–7° for 60% defects when using an 80 mm stem. This preliminary biomechanical finding offers a basis for exploring personalized implant design, while the realization of precision-based repair and improved prosthesis longevity requires further validation by multi-center clinical data, diverse patient anatomical models (e.g., differences in tibial size and medullary canal morphology), and *in vitro* experiments.These data need to be verified through multi-center clinical data and *in vitro* artificial bone experiments.

## 1 Introduction

Tibial plateau bone defect is a core challenge in revision total knee arthroplasty, and its repair outcome directly impacts the long-term stability of the prosthesis, the efficiency of joint mechanical conduction, and the medium-to-long-term prognosis of patients (Nadorf et al., 2017).In physiological conditions, the posterior tibial slope (PTS), as a key anatomical parameter in knee biomechanics, enables uniform load transmission to cancellous bone during knee flexion and extension by optimizing patellofemoral joint load distribution and balancing posterior cruciate ligament tension. Its optimal functional angle has been confirmed to stabilize within the range of 5°–7° (Chen et al., 2021).However, the occurrence of bone defects impairs the anatomical integrity of the proximal tibia: metaphyseal cancellous bone loss causes medullary canal axis deviation, and disruption of cortical bone continuity leads to abnormal mechanical conduction pathways. Traditional straight or fixed-angle extension stems fail to adapt to the complex medullary canal morphology after defect, easily triggering a cascade of problems—cortical bone stress concentration increases the risk of fracture, excessive cancellous bone micromotion inhibits osseointegration, uneven loading between the prosthesis and bone implant (titanium alloy Ti-6Al-4V sleeve, metal bone filler) accelerates metal fatigue or bone resorption, ultimately leading to prosthesis loosening, postoperative pain, and revision failure (Quilez et al., 2017).

Although existing studies have confirmed that physiological posterior tilt (5°–7°) can reduce the risk of polyethylene wear, and moderate-to-severe defects require matching the natural curvature of the distal medullary canal (average 6°) to reduce the 10-year loosening rate (Pourzal et al., 2020),critical gaps remain in clinical and basic research. First, bone defect severity is progressive (20% mild, 40% moderate, 60% severe), exerting differential effects on medullary canal morphology and stress transmission pathways. Whether the adaptation logic of posterior tilt angle dynamically adjusts with defect severity lacks systematic understanding. Second, extension stem length is coupled with the depth of the fixation zone (40 mm short stems rely on metaphyseal zone 2 fixation, while 80 mm long stems require synergistic diaphyseal zone 3 support). Quantitative analysis is lacking regarding whether there are essential differences in their coupling mechanisms with posterior tilt angle. Third, how the stress distribution and relative displacement of cortical bone, cancellous bone, prosthesis, and sleeve exhibit specific responses to the combination of “defect area-extension stem length-posterior tilt angle” has not been addressed by multi-structure synchronous analysis.

To address these gaps, this study established a multi-factor coupling model using the method of controlled variables: setting gradients of tibial plateau defect areas at 20%, 40%, and 60%, matching differences in extension stem lengths (40 mm vs. 80 mm), and covering posterior tilt angles of 0°, 3°, 5°, 7°, and 10°. It systematically and quantitatively analyzed the variation patterns of cortical bone stress, cancellous bone stress, prosthesis stress, bone implant stress, and relative displacement. The aim is to explore the potential synergistic optimization logic of “defect severity-extension stem length-posterior tilt angle,” provide a preliminary mechanical basis for investigating personalized implant design in complex bone defect repair, and lay a foundation for promoting the transformation of knee revision from “empirical repair” to a “precision morphology-mechanics matching” model. Notably, the study only provides a hypothetical reference for the selection of posterior tilt angles of extension stems based on finite element analysis, and this reference can only be gradually applied to clinical practice after validation by multi-center clinical data, diverse patient anatomical models (e.g., differences in tibial size and medullary canal morphology), and *in vitro* experiments—with the ultimate goal of improving the medium-to-long-term survival rate of prostheses and patients’ functional outcomes.

## 2 Materials and methods

### 2.1 Establishment of tibial plateau bone defect models

**FIGURE 1:**
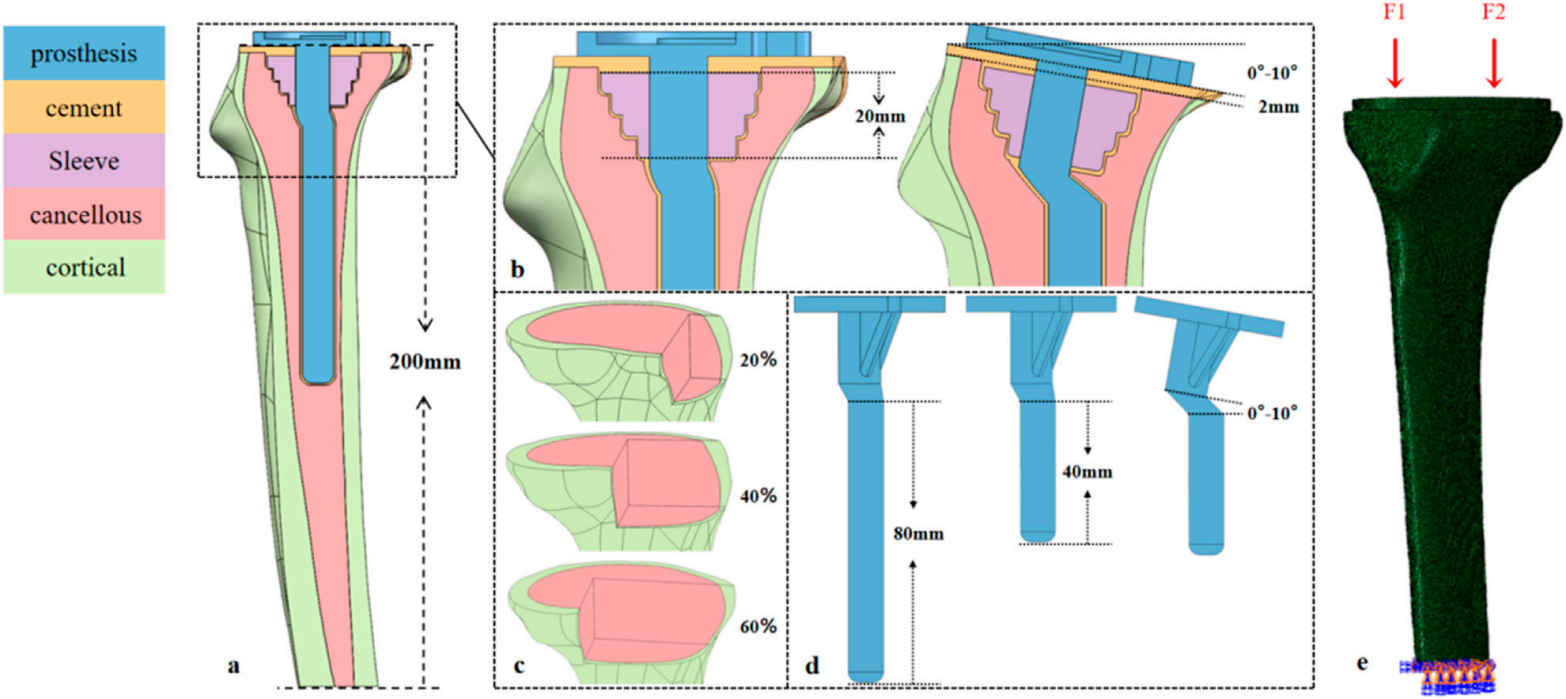
Design of the tibial prosthesis-bone integration model: Defect, sagittal tilt, and biomechanical loading. **(a)** Tibial model (height ≈200 mm) covering the proximal metaphysis and distal diaphysis. **(b)** 20-mm-long bone filler (sleeve) in the metaphyseal region; sagittal tilt gradients of the tibial plateau (0°, 3°, 5°, 7°, 10°); and a 2-mm-thick PMMA cement layer between the prosthesis and bone. **(c)** Area defects (20%, 40%, 60%) in the tibial plateau region. **(d)** Tibial prostheses with 40-mm/80-mm prosthetic stem, where the prosthesis and stem are synchronously tilted (0°–10°) to match the plateau sagittal angle. **(e)** Medial (F_1_) and lateral (F_2_) physiological loads on the tibial plateau; full-degree-of-freedom constraint at the distal tibia.

A 24-year-old healthy male volunteer (height: 185 cm, weight: 70 kg) was recruited. After signing an informed consent form, knee joint scanning was performed using a Siemens SOMATOM Definition AS 128-slice CT scanner (tube voltage: 70 kV). DICOM images were imported into Mimics 21.0, and a three-dimensional model was constructed by distinguishing cortical bone from cancellous bone based on grayscale values; subsequent surface repair and smoothing were performed using Geomagic Wrap 2021. Three types of bone defect models were established in SOLIDWORKS 2021 (Figure 1c): with a depth of 22 mm and defect areas of 20%, 40%, and 60%, respectively. Parameters for tibial prosthesis implantation were set as follows: the proximal cutting surface was perpendicular to the mechanical axis of the coronal plane; posterior tilt angles were 0°, 3°, 5°, 7°, and 10° (defined as the angle between the sagittal cutting surface and the anatomical axis); and extension stems matching the posterior tilt angles (40 mm/80 mm) were designed (Figure 1d). A 2.0-mm bone cement layer was used to fix the tibial tray and Sleeve, with the defect area defined as the bone cement-filled region (Figure 1b). The model was meshed with C3D4 tetrahedral elements in Hypermesh, with a global mesh size of 1 mm (Zheng et al., 2020), and then imported into Abaqus for analysis.

### 2.2 Model parameters and grouping

All materials were assumed to be continuous, isotropic linear elastic materials, with mechanical parameters as shown in Table 1 (Brihault et al., 2016; Zheng et al., 2020). Variables were defined as follows: D denotes defect area (with 20%, 40%, and 60% corresponding to D20, D40, and D60, respectively); P denotes posterior tilt angle (with 0°, 3°, 5°, 7°, and 10° corresponding to P0, P3, P5, P7, and P10, respectively); and E denotes extension stem length (with 40 mm and 80 mm corresponding to E40 and E80, respectively). All models were cross-combined according to the factor level combination of “defect area × extension stem length × posterior tilt angle”, resulting in a total of 3 (D20/D40/D60) × 2 (E40/E80) × 5 (P0/P3/P5/P7/P10) = 30 cases.

**TABLE 1:** Mechanical properties of different parts of the model.

Component	Material	Young’s modulus E (MPa)	Poisson’s ratio(v)
Prosthesis	Ti-6Al-4V	110,000	0.3
Sleeve			
Cement	PMMA	2270	0.46
Cortical bone	Cortical bone	17,000	0.3
Cancellous bone	Cancellous bone	700	0.3

### 2.3 Loading conditions

Based on the biomechanical characteristics of knee joint gait, the medial plateau bears approximately 60% (F1) and the lateral plateau 40% (F2) of the axial load during the mid-gait phase (Rasnick et al., 2016),Therefore, an axial load of 3.5 times the body weight (2450 N) was applied to the 70 kg subject, acting along the negative direction of the Z-axis in the model coordinate system to simulate the direction of the tibial mechanical axis. Contact relationships were defined as follows: fully bonded contact was adopted for the cortical bone-cancellous bone and cancellous bone-bone cement interfaces; a general contact algorithm was used for the bone cement-prosthesis and prosthesis-sleeve interfaces with a friction coefficient of 0.1 (Frehill and Crocombe, 2019); All degrees of freedom of nodes at 200 mm from the distal tibia were constrained to simulate physiological diaphyseal support (Figures 1a,e).

### 2.4 Grid convergence study

A representative experimental group (e.g., 40% bone defect, 80 mm stem, 7° retroversion) was selected as the validation model. Five sets of mesh schemes with different global element sizes (0.5mm, 1mm, 1.5mm, 2mm, 2.5 mm) were generated, while keeping the local mesh refinement strategy consistent (e.g., 0.3 mm refinement at the bone-prosthesis interface). The key output parameters of each scheme (peak stress of cortical bone, peak stress of cancellous bone, and bone-prosthesis micromotion) were calculated. The relative error method was used to evaluate convergence: when the element size was reduced from 1 mm to 0.5mm, if the relative errors of all key parameters were less than 5%, it was confirmed that the global mesh size of 1 mm (used in the original study) had converged and had high computational efficiency.

## 3 Results

### 3.1 Definition of micromotion

The relative micromotion between bone and prosthesis is defined as the relative displacement between the tibial prosthesis and the tibial bone tissue. Specifically, it is calculated by measuring the displacement difference between adjacent nodes at the bone-prosthesis interface (one located on the surface of the bone tissue and one on the surface of the prosthesis). The displacement components in the sagittal plane (anteroposterior direction), coronal plane (medial-lateral direction), and axial direction (superior-inferior direction) are recorded, among which the sagittal plane displacement is the core analysis index (directly related to the posterior tilt angle design).

### 3.2 In the 20% bone defect model

**FIGURE 2:**
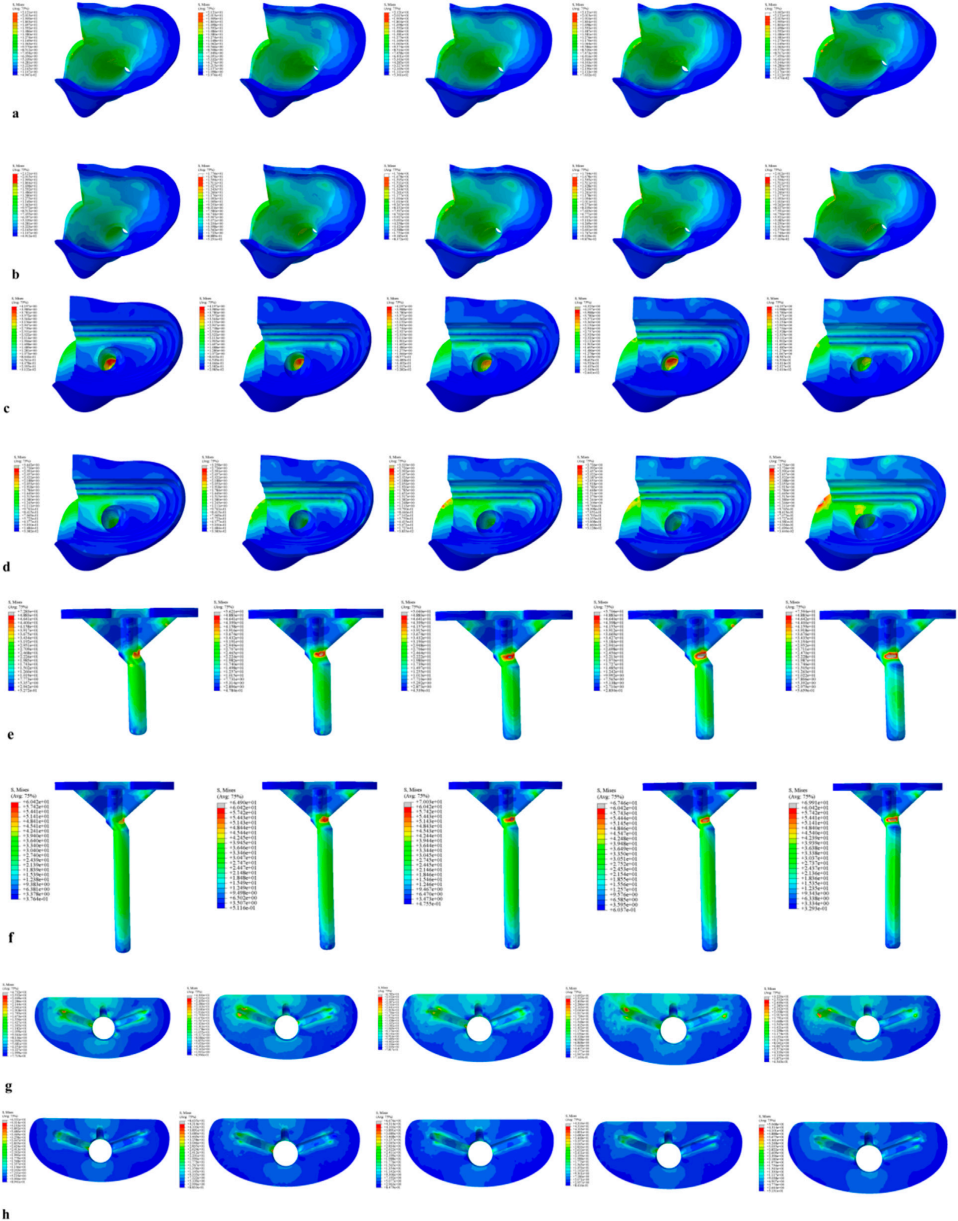
Von Mises stress on each model component under a 2450 N load. **(a)** Von Mises stress of cortical bone with the 40 mm extension stem. **(b)** Von Mises stress of cortical bone with the 80 mm extension stem. **(c)** Von Mises stress of cancellous bone with the 40 mm extension stem. **(d)** Von Mises stress of cancellous bone with the 80 mm extension stem. **(e)** Von Mises stress of the 40 mm extension stem. **(f)** Von Mises stress of the 80 mm extension stem. **(g)** Von Mises stress of the bone implant (sleeve) with the 40 mm extension stem. **(h)** Von Mises stress of the bone implant (sleeve) with the 80 mm extension stem.

**FIGURE 3:**
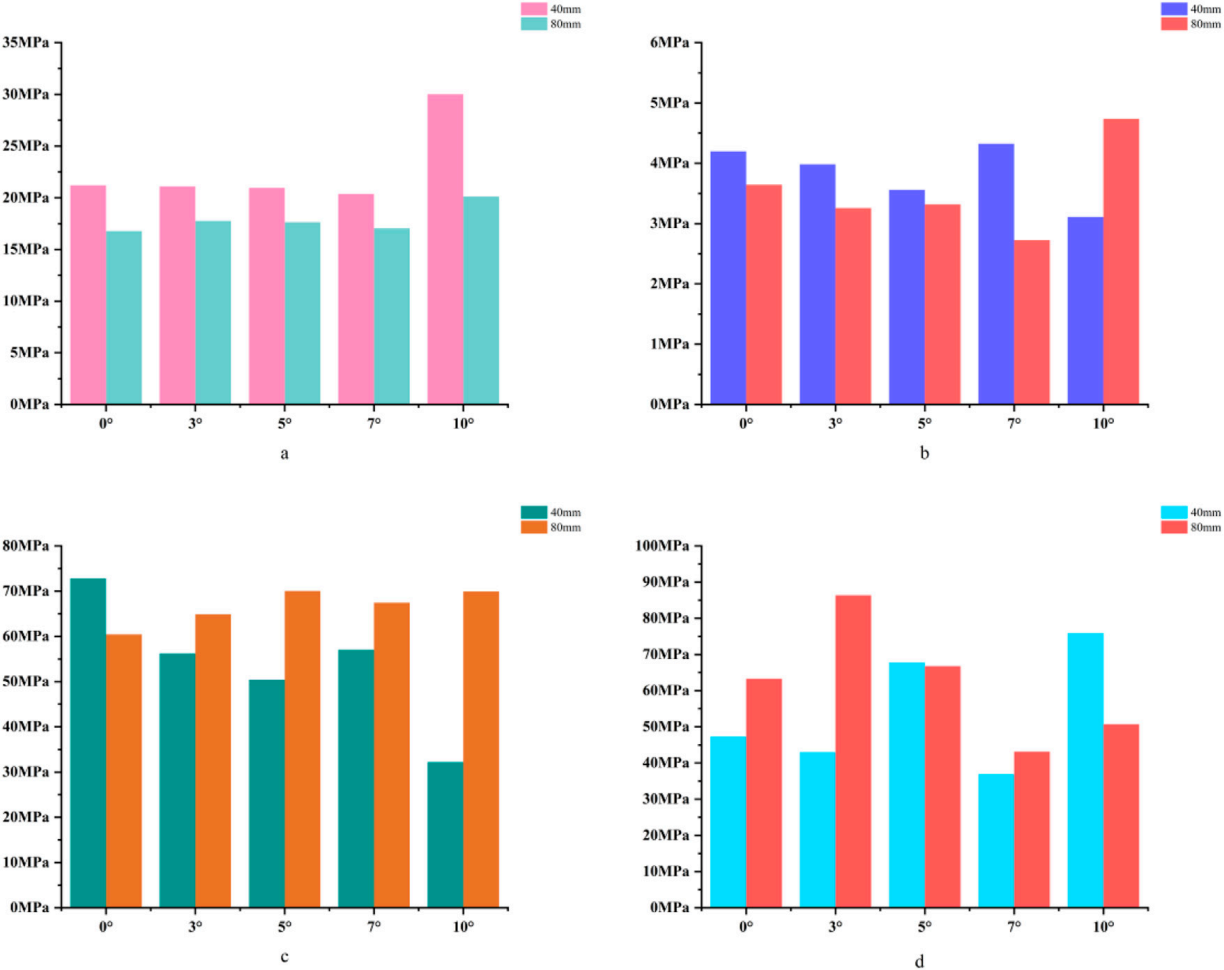
**(a)** Bar chart of maximum stress in cortical bone. **(b)** Bar chart of maximum stress in cancellous bone. **(c)** Bar chart of maximum stress in the prosthesis. **(d)** Bar chart of maximum stress in the bone implant (sleeve).

**FIGURE 4:**
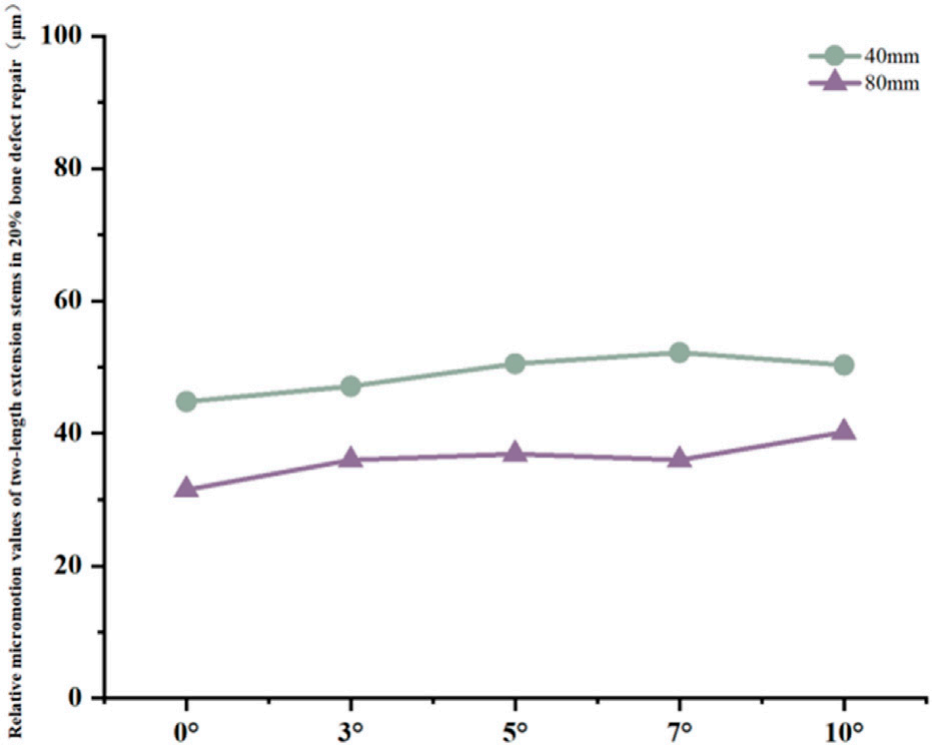
Line graph of relative micromotion for two extension stems of different lengths in repairing 20% bone defects.

For the 40 mm extension stem, the peak cortical bone stress reached 30.02 MPa at a 10° posterior tilt, representing an increase of 47.4% compared to that at 7°; in contrast, the corresponding value for the 80 mm extension stem at 10° posterior tilt was 20.12 MPa, an increase of 19.9% compared to that at 0° (Figures 2a,b) (Figure 3a).For the 40 mm extension stem, the peak cancellous bone stress was the lowest at 3.110 MPa at 10° posterior tilt, which was a 39% increase compared to that at 7°; for the 80 mm extension stem, the peak cancellous bone stress increased from 2.726 MPa at 7° posterior tilt to 4.736 MPa at 10° posterior tilt, showing an increase of 73.7% (Figures 2c,d) (Figure 3b).In the case of the 40 mm extension stem at 10° posterior tilt, the prosthesis stress decreased from 72.83 MPa at 0° to 32.2 MPa, with a reduction of 55.8%; while the peak stress of the bone implant (sleeve) increased from 47.32 MPa at 0° to 75.94 MPa at 10°, an increase of 60.5%. For the 80 mm extension stem at 10° posterior tilt, the prosthesis stress increased from 60.42 MPa at 0° to 69.91 MPa, with an increase of 15.7%; whereas the peak stress of the sleeve decreased from 63.81 MPa at 0° to 50.68 MPa at 10°, a reduction of 24.9% (Figures 2e–h) (Figures 3c,d).The relative micromotion values in all cases were lower than the critical threshold of 150 μm for sseointegration. For the 40 mm extension stem, the micromotion value was the lowest at 44.8 μm at 0° posterior tilt and increased to the highest value of 52.2 μm at 7°. For the 80 mm extension stem, the micromotion value was the lowest at 31.5 μm at 0° posterior tilt and increased to the highest value of 40.2 μm at 10°, but remained consistently lower than the corresponding values of the 40 mm extension stem under the same conditions (Figure 4).

### 3.3 In the 40% bone defect model

**FIGURE 5:**
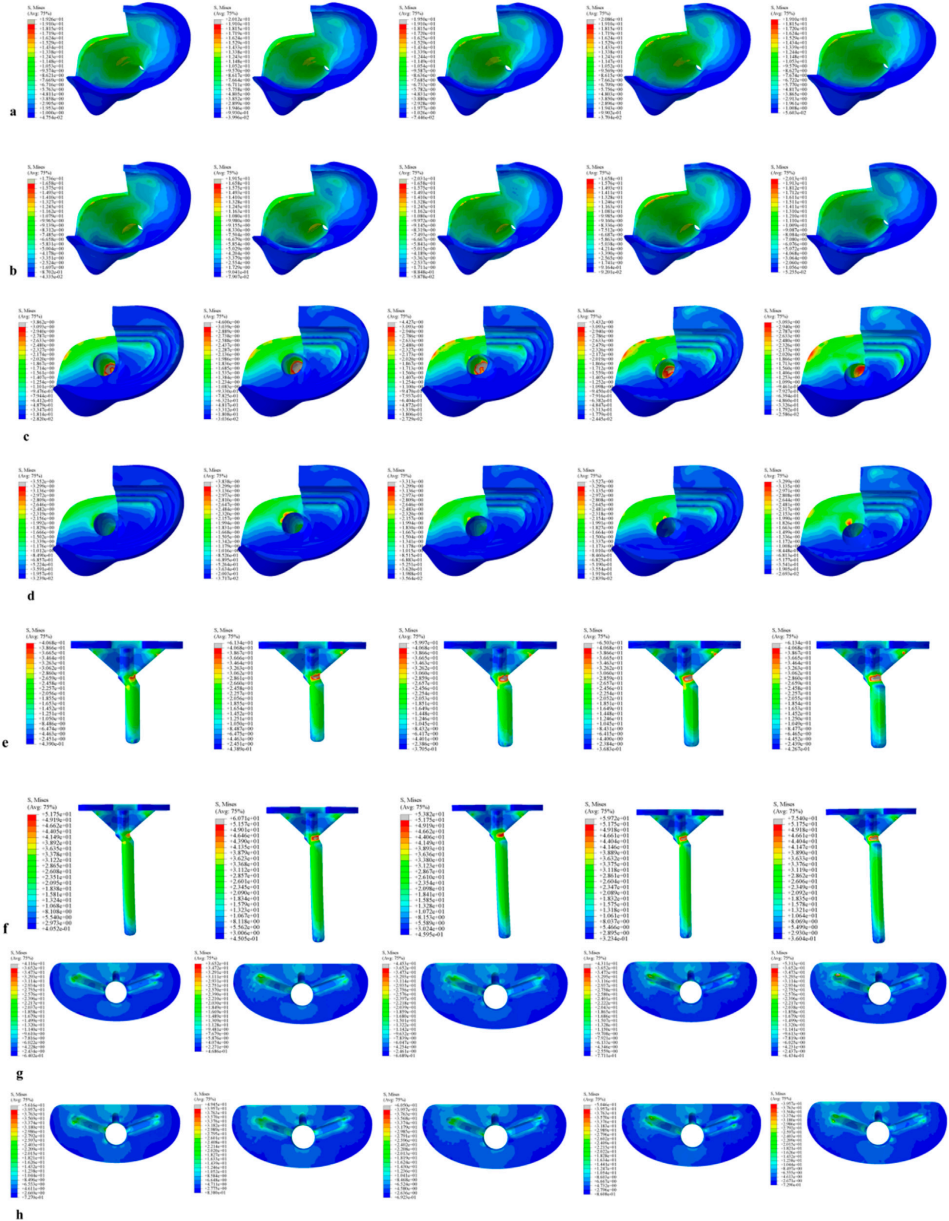
Von Mises stress on each model component under a 2450 N load. **(a)** Von Mises stress of cortical bone with the 40 mm extension stem. **(b)** Von Mises stress of cortical bone with the 80 mm extension stem. **(c)** Von Mises stress of cancellous bone with the 40 mm extension stem. **(d)** Von Mises stress of cancellous bone with the 80 mm extension stem. **(e)** Von Mises stress of the 40 mm extension stem. **(f)** Von Mises stress of the 80 mm extension stem. **(g)** Von Mises stress of the bone implant (sleeve) with the 40 mm extension stem. **(h)** Von Mises stress of the bone implant (sleeve) with the 80 mm extension stem.

**FIGURE 6:**
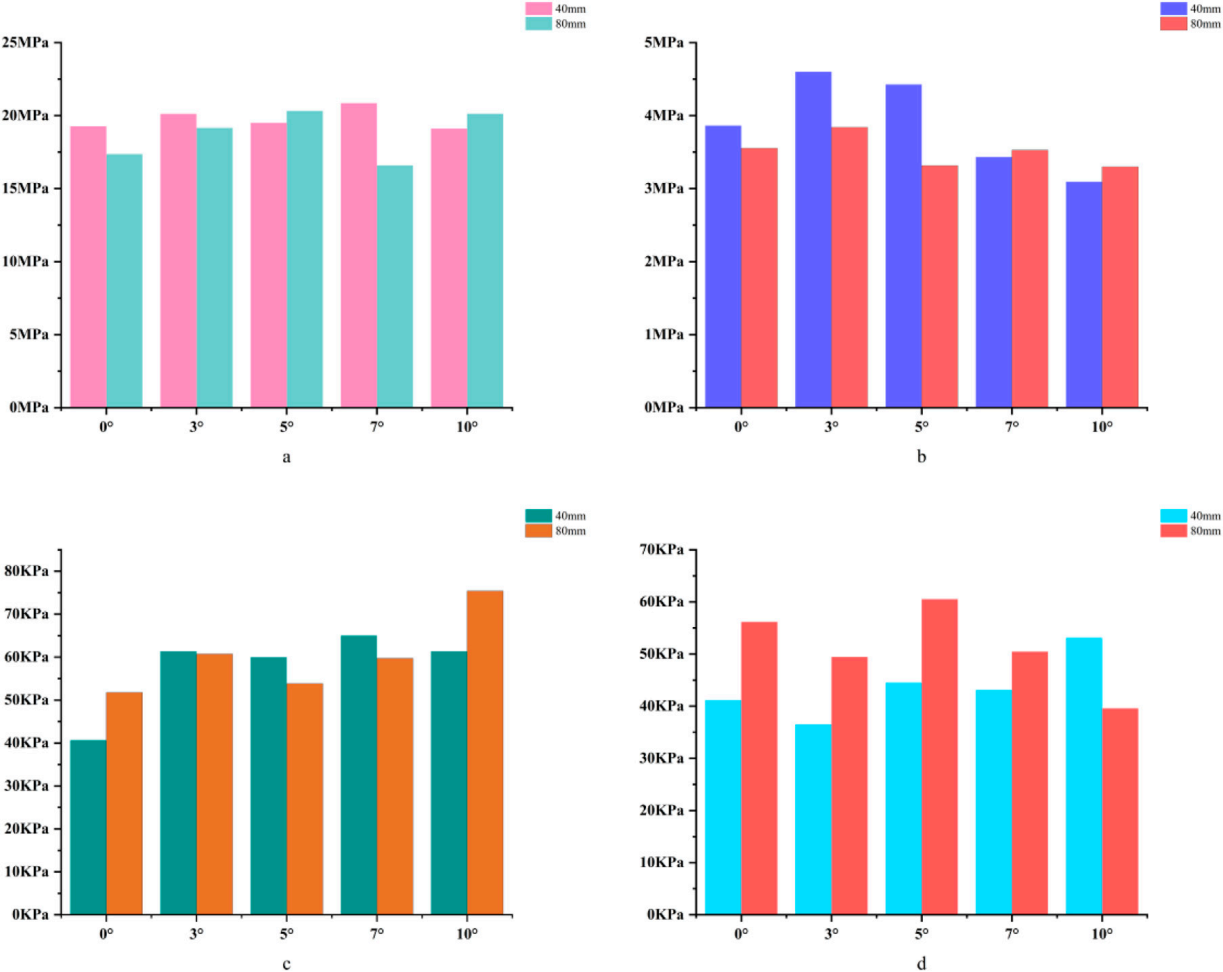
**(a)** Bar chart of maximum stress in cortical bone. **(b)** Bar chart of maximum stress in cancellous bone. **(c)** Bar chart of maximum stress in the prosthesis. **(d)** Bar chart of maximum stress in the bone implant (sleeve).

**FIGURE 7:**
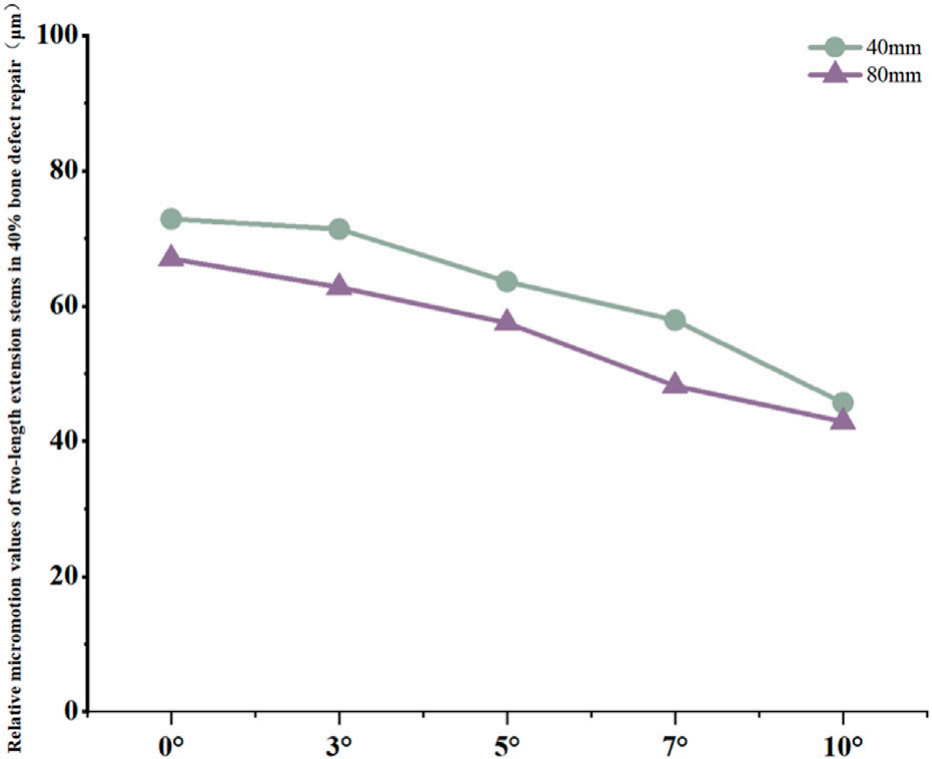
Line graph of relative micromotion values for two extension stems of different lengths in repairing 40% bone defects.

For the 40 mm extension stem, the cortical bone stress was the lowest at 19.1 MPa at 10° posterior tilt, showing an 8.4% decrease compared to that at 7°; in contrast, the corresponding value for the 80 mm extension stem at the same tilt angle was 20.13 MPa, an increase of 21.4% compared to that at 7° (Figures 5a,b) (Figure 6a).For the 40 mm extension stem, the peak cancellous bone stress dropped to the lowest value of 3.093 MPa at 10° posterior tilt, which was a 48.7% increase compared to that at 3°; for the 80 mm extension stem, the peak cancellous bone stress decreased from 3.838 MPa at 3° posterior tilt to 3.299 MPa at 10° posterior tilt, with a reduction of 16.3% (Figures 5c,d) (Figure 6b).For the 40 mm extension stem, the prosthesis stress increased from 40.68 MPa at 0° to 65.03 MPa at 7°, with an increase of 59.9%; while the peak stress of the bone implant (sleeve) decreased from 36.52 MPa at 3° to 53.13 MPa at 10°, showing a reduction of 45.5%. For the 80 mm extension stem, the prosthesis stress increased from 51.75 MPa at 0° posterior tilt to 75.40 MPa at 10°, with an increase of 45.7%; whereas the peak stress of the sleeve decreased from 56.16 MPa at 0° to 39.57 MPa at 10°, a reduction of 29.5% (Figures 5e–h) (Figures 6c,d).Relative micromotion values in all cases were lower than the critical threshold of 150 μm for osseointegration, and the overall values decreased with increasing posterior tilt angles. For the 40 mm extension stem, the micromotion value was the lowest at 45.7 μm at 10° posterior tilt and the highest at 72.9 μm at 0°. For the 80 mm extension stem, the micromotion value was the lowest at 42.9 μm at 10° posterior tilt and the highest at 67 μm at 0°, but remained consistently lower than the corresponding values of the 40 mm extension stem under the same conditions (Figure 7).

### 3.4 In the 60% bone defect model

**FIGURE 8:**
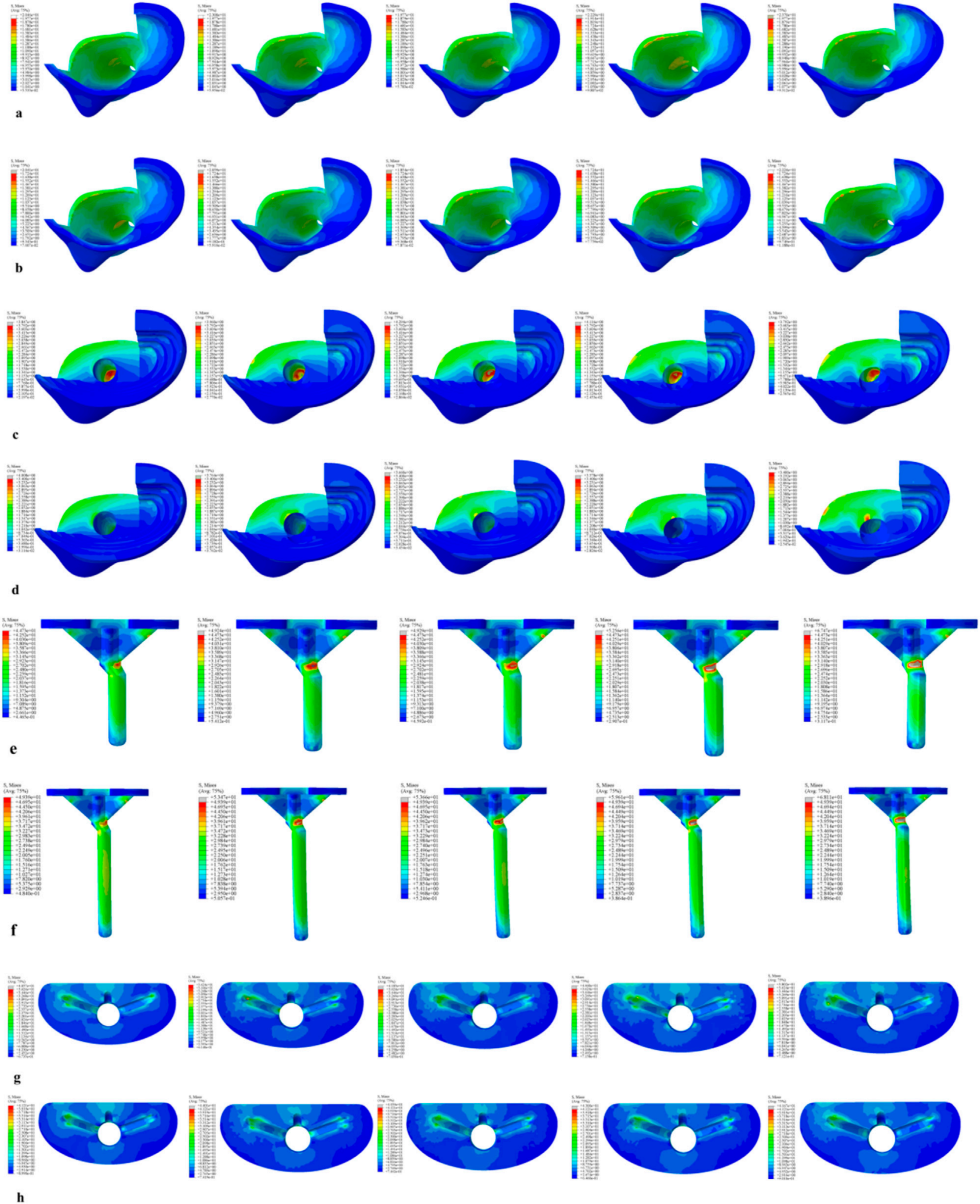
Von Mises stress on each model component under a 2450 N load. **(a)** Von Mises stress of cortical bone with the 40 mm extension stem. **(b)** Von Mises stress of cortical bone with the 80 mm extension stem. **(c)** Von Mises stress of cancellous bone with the 40 mm extension stem. **(d)** Von Mises stress of cancellous bone with the 80 mm extension stem. **(e)** Von Mises stress of the 40 mm extension stem. **(f)** Von Mises stress of the 80 mm extension stem. **(g)** Von Mises stress of the bone implant (sleeve) with the 40 mm extension stem. **(h)** Von Mises stress of the bone implant (sleeve) with the 80 mm extension stem.

**FIGURE 9:**
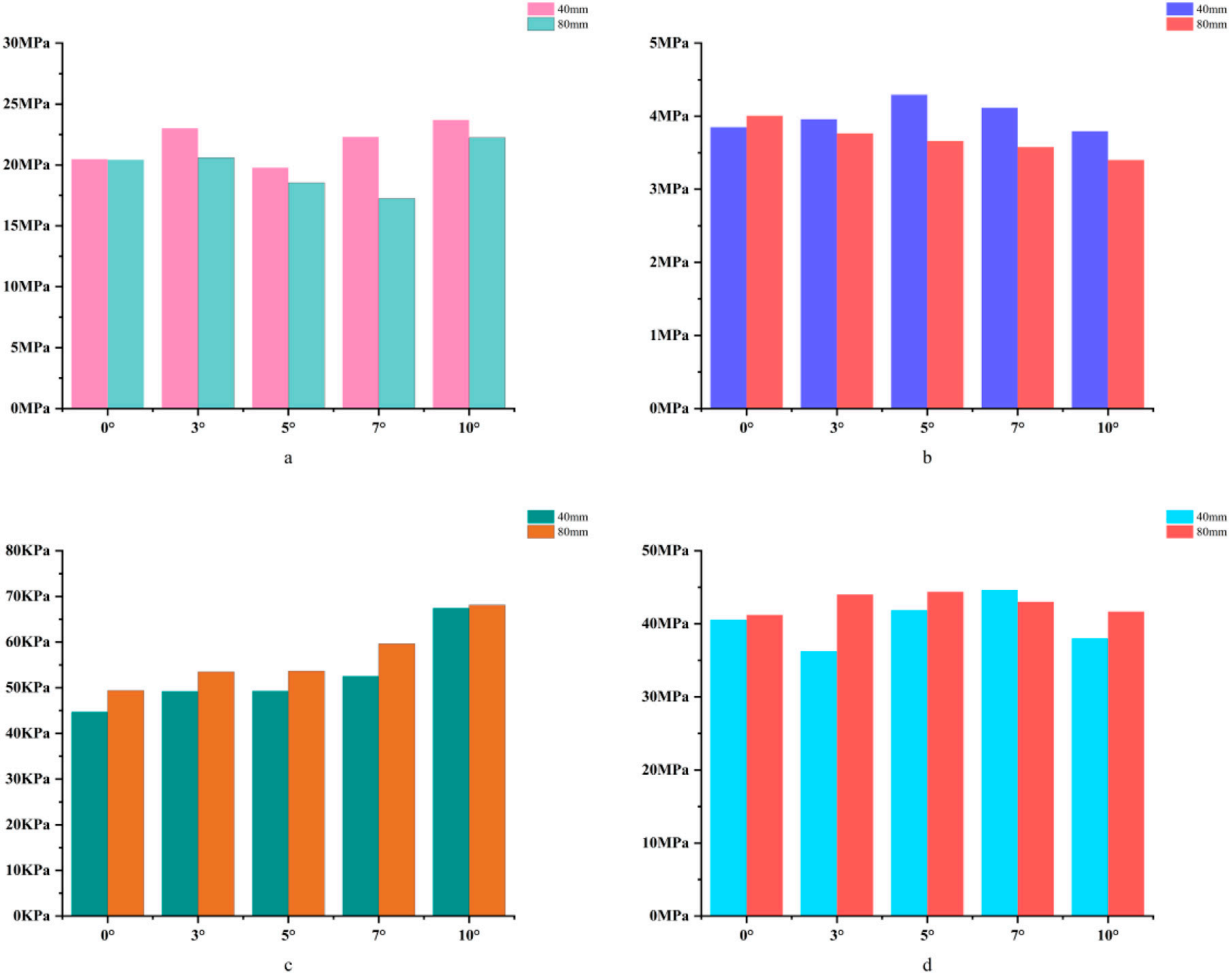
**(a)** Bar chart of maximum stress in cortical bone. **(b)** Bar chart of maximum stress in cancellous bone. **(c)** Bar chart of maximum stress in the prosthesis. **(d)** Bar chart of maximum stress in the bone implant (sleeve).

For the 40 mm extension stem, the cortical bone stress was the lowest at 19.77 MPa at 5° posterior tilt, while the corresponding value for the 80 mm extension stem at the same tilt angle (5°) was 18.54 MPa (Figures 8a,b) (Figure 9a).For the 40 mm extension stem, the peak cancellous bone stress dropped to the lowest value of 3.792 MPa at 10° posterior tilt, which was a 13.2% increase compared to that at 5°; for the 80 mm extension stem, the peak cancellous bone stress decreased from 4.008 MPa at 0° posterior tilt to 3.4 MPa at 10° posterior tilt, with a reduction of 17.9% (Figures 8c,d) (Figure 9b).For the 40 mm extension stem, the prosthesis stress increased from 44.73 MPa at 0° to 67.47 MPa at 10°, showing an increase of 50.8%; the stress of the bone implant (sleeve) was the lowest at 36.24 MPa at 3° and the highest at 44.6 MPa at 7°. For the 80 mm extension stem, the prosthesis stress increased from 41.21 MPa at 0° posterior tilt to 44.39 MPa at 5°, with an increase of 7.7%; the peak stress of the sleeve was 44.39 MPa at 5° (Figures 8e–h) (Figures 9c,d).

**FIGURE 10:**
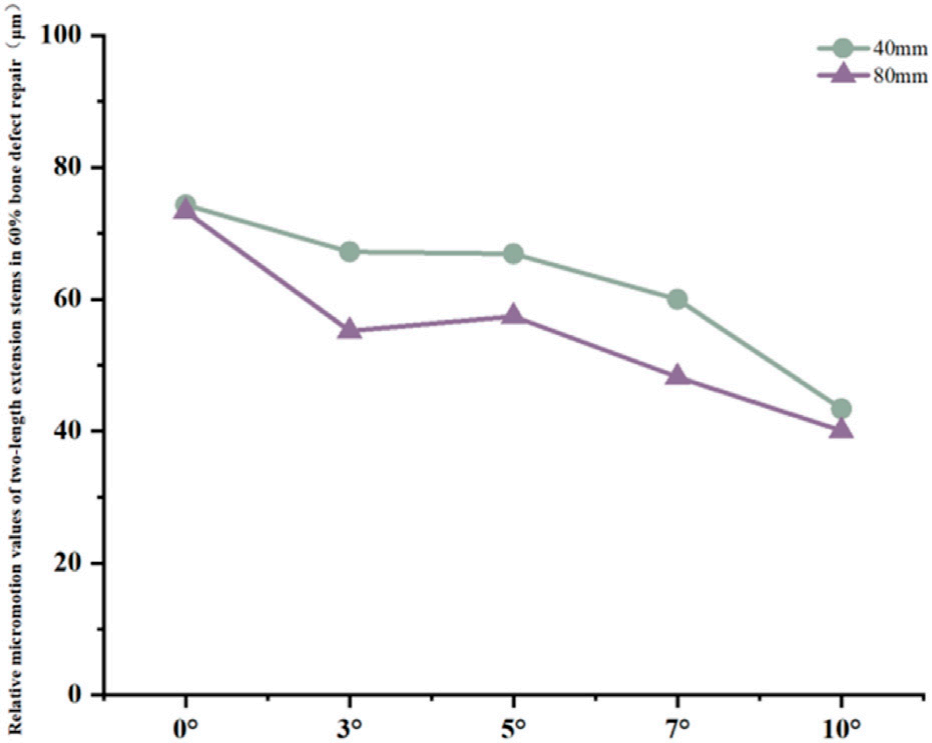
Line graph of relative micromotion values for two extension stems of different lengths in repairing 60% bone defects.

Relative micromotion values in all cases were lower than the critical threshold of 150 μm for osseointegration, and the overall values decreased with increasing posterior tilt angles. For the 40 mm extension stem, the micromotion value was the lowest at 43.4 μm at 10° posterior tilt and the highest at 74.3 μm at 0°. For the 80 mm extension stem, the micromotion value was the lowest at 40.1 μm at 10° posterior tilt and the highest at 73.3 μm at 0°, but remained consistently lower than the corresponding values of the 40 mm extension stem under the same conditions (Figure 10).

## 4 Discussion

Posterior tibial slope (PTS) is a key parameter influencing implant stability and patient comfort in knee arthroplasty (Whiteside and Amador, 1988),For bone defect repair, the posterior tilt design of extension stems must simultaneously match physiological anatomy and defect characteristics. This study focused on the mechanical performance of 40 mm and 80 mm extension stems at posterior tilt angles of 0°, 3°, 5°, 7°, and 10° under different tibial plateau defect areas (20%, 40%, 60%), revealing the synergistic optimization laws of “defect severity-extension stem length-posterior tilt angle”. Results showed that posterior tilt extension stems can reduce the risk of cortical contact by matching the natural morphology of the medullary canal, and their angle selection needs to be dynamically adjusted with defect area. This finding is highly consistent with existing anatomical and biomechanical studies, providing experimental evidence for personalized strategies in complex bone defect repair.

Relative micromotion at the bone-prosthesis interface is a critical factor affecting osseointegration. Micromotion less than 150 μm facilitates callus formation and mechanical integration; exceeding this threshold tends to form fibrous connective tissue, leading to prosthesis loosening (Pilliar et al., 1986). In this study, relative micromotion in all cases was below 150 μm, suggesting that posterior tilt-matched extension stems have a mechanical basis for promoting osseointegration, but with significant interaction effects: in 20% defects, micromotion of 40 mm and 80 mm stems increased by 12.3% and 27.6% respectively with increasing angle, presumably due to excessive posterior tilt causing the extension stem to deviate from the cancellous bone center, which is consistent with the theory that “excessive posterior tilt alters tibiofemoral pressure distribution” (Angerame, 2019)。Stress concentration in the anterior tibial cortical bone is a potential cause of periprosthetic pain (Simpson et al., 2011).:In 20% defects, the cortical stress of 40 mm and 80 mm stems increased by 41.5% and 19.9% respectively with increasing angle, suggesting that excessive posterior tilt may cause “cortical impingement”, which is consistent with the conclusion that “prosthesis inclination alters stress conduction pathways” (Yuan, 2023); In 40% and 60% defects, the cortical stress of 80 mm stems increased by 8.9%–15.9% with increasing posterior tilt angle, indicating the need to warrant caution regarding stress risks associated with extreme posterior tilt in long stems. For cancellous bone stress, in most cases of 40% and 60% defects, stress decreased with increasing angle (e.g., a 20.0% reduction in 40 mm stems for 40% defects); however, in 20% defects, cancellous bone stress increased by 30.0% at 10° posterior tilt for 80 mm stems, suggesting that excessive posterior tilt in long stems for low-severity defects requires caution. Stress transfer between the prosthesis and sleeve reflects the load distribution mechanism: in 20% defects, with 40 mm stems at 10° posterior tilt, prosthesis stress decreased by 55.8% while sleeve stress increased by 60.5%; in 40% and 60% defects, with 80 mm stems at 10° posterior tilt, prosthesis stress increased significantly (45.7%–37.9%) whereas sleeve stress decreased. It is hypothesized that after long stems enhance stability, the prosthesis bears more load, necessitating attention to fatigue life. Notably, this study has certain inherent limitations. Specifically, all tissues involved—including cortical bone, cancellous bone, the prosthesis, and the sleeve—were modeled as continuous, isotropic linear elastic materials. This simplification overlooks the anisotropic properties of natural bone (e.g., the stiffness of cortical bone along the longitudinal axis of the tibia is significantly higher than its transverse stiffness) as well as the viscoelastic behavior of polymethyl methacrylate (PMMA) bone cement. Nevertheless, such simplifications are widely accepted and applied in preliminary biomechanical parameterization studies of knee arthroplasty. Critically, they do not exert a significant influence on the qualitative trend of stress distribution under the coupling effect of “defect severity-stem length-posterior tilt angle”—the core focus of this research. For future investigations, an anisotropic bone model will be constructed based on CT grayscale values to more accurately replicate the mechanical characteristics of natural bone, thereby further enhancing the precision of biomechanical simulations. Another limitation is related to the loading and boundary condition settings. We acknowledge that the current study only adopted a single 2450N axial load (combined with distal tibial fixation), which fails to fully simulate the physiological characteristics of multi-axial loads (e.g., shear forces, bending moments), muscle-driven loads, and flexion angle-dependent loads in revision total knee arthroplasty (revision TKA). To address this deficiency, future research will supplement multi-axial load components (such as sagittal shear forces and coronal bending moments) and simulate different knee flexion angles (0°, 30°, and 60°, corresponding to functional scenarios including standing, walking, and stair climbing). Meanwhile, the distal tibial boundary conditions will be optimized to better approximate the physiological support state, and the effects of the aforementioned load conditions on the stress distribution and micromotion characteristics of the bone-prosthesis interface will be analyzed in depth.A third limitation concerns the lack of in-depth data analysis to verify result reliability and variable coupling mechanisms. The current study did not conduct data uncertainty analysis (e.g., calculation of parameter variation coefficients), sensitivity testing (e.g., evaluation of how perturbations in key material parameters affect simulation results), or factor regression analysis (e.g., quantitative assessment of the main effects and interaction effects of defect severity, stem length, and posterior tilt angle). These analyses are essential for quantifying the robustness of the study’s conclusions and clarifying the statistical significance of the synergistic coupling between core variables. In subsequent research, these analytical approaches will be supplemented: uncertainty analysis will help characterize the variability of results under parameter fluctuations; sensitivity testing will identify the material parameters that most strongly influence biomechanical outcomes; and factor regression analysis will quantitatively delineate the contribution of each variable and their interactive effects on bone-prosthesis stress and micromotion. This will further validate the reliability of the current findings and strengthen the scientific basis for the proposed “defect-stem-tilt” optimization strategy.These limitations do not undermine the core conclusion of this study regarding the synergistic optimization of “defect severity-extension stem length-posterior tilt angle”; instead, they provide clear directions for further refining the biomechanical simulation system of revision TKA, which is expected to provide more comprehensive and practical mechanical evidence for personalized implant design in clinical practice.

## 5 Conclusion

This study constructed a multi-factor coupling model incorporating different tibial plateau defect areas (20%, 40%, 60%), extension stem lengths (40 mm, 80 mm), and posterior tilt angles (0°, 3°, 5°, 7°, 10%). It systematically analyzed the stress distribution and relative micromotion characteristics at the bone-prosthesis interface, revealing the synergistic optimization laws of “defect severity-extension stem length-posterior tilt angle”.

The results demonstrated that relative micromotion at the bone-prosthesis interface in all cases was below the 150 μm critical threshold for osseointegration, confirming that extension stems with posterior tilt design possess a mechanical basis for promoting osseointegration. This is attributed to the posterior tilt’s ability to match the natural or defect-altered morphology of the tibial medullary canal, thereby reducing the risk of “cortical impingement” (excessive contact between the stem and anterior cortical bone) that plagues traditional straight stems. However, stress distribution and micromotion characteristics exhibited significant defect dependence:

In 20% mild defects, the medullary canal retains most of its physiological morphology (close to the 5°–7° natural posterior tibial slope). For 40 mm short stems, a posterior tilt angle of ≤7° is recommended to avoid cortical stress concentration—at 10° tilt, the peak cortical stress reaches 30.02 MPa, a 47.4% increase compared to 7° (Figure 3a). Although 80 mm long stems show lower micromotion, their cancellous bone stress at 10° tilt increases by 73.7% compared to 7° (Figure 3b), making this angle risky and requiring cautious selection.

In 40% moderate defects, bone loss causes significant medullary canal distortion, and 80 mm long stems (relying on synergistic diaphyseal-metaphyseal fixation) perform better in stability. Increasing the posterior tilt to 7°–10° can reduce micromotion by 37.3% (from 67 μm at 0° to 42.9 μm at 10°, Figure 7); while cortical stress at 10° (20.13 MPa) increases by 21.4% compared to 7° (16.5 MPa, Figure 6a), the diaphyseal fixation of the long stem disperses the load, preventing catastrophic stress concentration.

In 60% severe defects, medullary canal damage is more severe, and a balance between micromotion control and stress distribution is critical. For 80 mm long stems, a 5°–7° posterior tilt achieves this balance: it reduces micromotion by 45.3% (from 73.3 μm at 0° to 40.1 μm at 10°, Figure 10), and the cortical stress at 5° (18.54 MPa) is lower than the 22.3 MPa at 10° (Figure 9a), while avoiding the excessive prosthesis load increase (45.7% higher than 0°, Figure 6c) caused by 10° tilt.

In summary, for tibial plateau bone defect repair, the selection of extension stem posterior tilt angle needs to be dynamically adjusted with defect severity and synergistically optimized with extension stem length—essentially, posterior tilt extension stems reduce cortical contact risk by matching medullary canal morphology (whether physiological or defect-altered), and their optimal angle is determined by the changes in medullary load-bearing capacity and morphology induced by bone defects. This study provides a precise biomechanical basis for personalized implant design in complex bone defect repair, facilitating the transformation of knee revision surgery from “empirical repair” to a “precision morphology-mechanics matching” model, thereby improving the medium-to-long-term survival rate of prostheses and patients’ functional outcomes.

## Data Availability

The original contributions presented in the study are included in the article/supplementary material, further inquiries can be directed to the corresponding authors.
